# Bio-stimulants enhance cold tolerance in tobacco through antioxidant defense and photosynthetic regulation

**DOI:** 10.3389/fmicb.2026.1884028

**Published:** 2026-07-20

**Authors:** Washu Dev, Kexin Yang, Siqi Ma, Jinfeng Zhang, Ping Zou, Yanxiu Li, Siyao Xie, Changliang Jing, Yuande Liu, Zhiming Hu

**Affiliations:** 1 Tobacco Research Institute of the Chinese Academy of Agricultural Sciences, Qingdao, China; 2 Baoshan Branch, Tobacco Company, Baoshan, Yunnan, China; 3 Linyi Branch, Tobacco Company, Linyi, Shandong, China

**Keywords:** antioxidant, bio-stimulants, cold stress, photosynthetic, tobacco (*Nicotiana tabacum* L.)

## Abstract

Cold stress (CS) is a significant obstacle in tobacco (*Nicotiana tabacum* L.) farming, significantly affecting plant development, photosynthetic activity, and cellular redox balance. In recent decades, bio-stimulants have created environmentally friendly substances that make the plant resistant to abiotic stresses, such as cold stress. The recent developments highlight bio-stimulants as a sustainable solution to improve cold stress tolerance in tobacco production. These substances promote plant growth, thereby increasing plant resilience to unfavorable temperature conditions. This review assesses the role of bio-stimulants in improving cold stress tolerance in tobacco, focusing on physiological, biochemical, and molecular responses. It summarizes the effects of various bio-stimulants on plant growth, antioxidant defense systems, and photosynthetic performance under low-temperature conditions. The enhancement of enzymatic antioxidants and non-enzymatic antioxidants by bio-stimulants helps overcome oxidative damage. Evidence in molecular biology studies to understand bio-stimulant-mediated regulation of stress responsive genes is also critically discussed in order to understand the role bio-stimulants play in enhancing the genetic potential of tobacco to cold stress. This review presents an integrated scheme of the multifarious functions of bio-stimulants in improving cold stress tolerance of tobacco. It also highlights existing knowledge gaps and provides research directions on how to explore efficient, sustainable, and climate resilient tobacco production systems in the future.

## 1 Introduction

**Figure 1:**
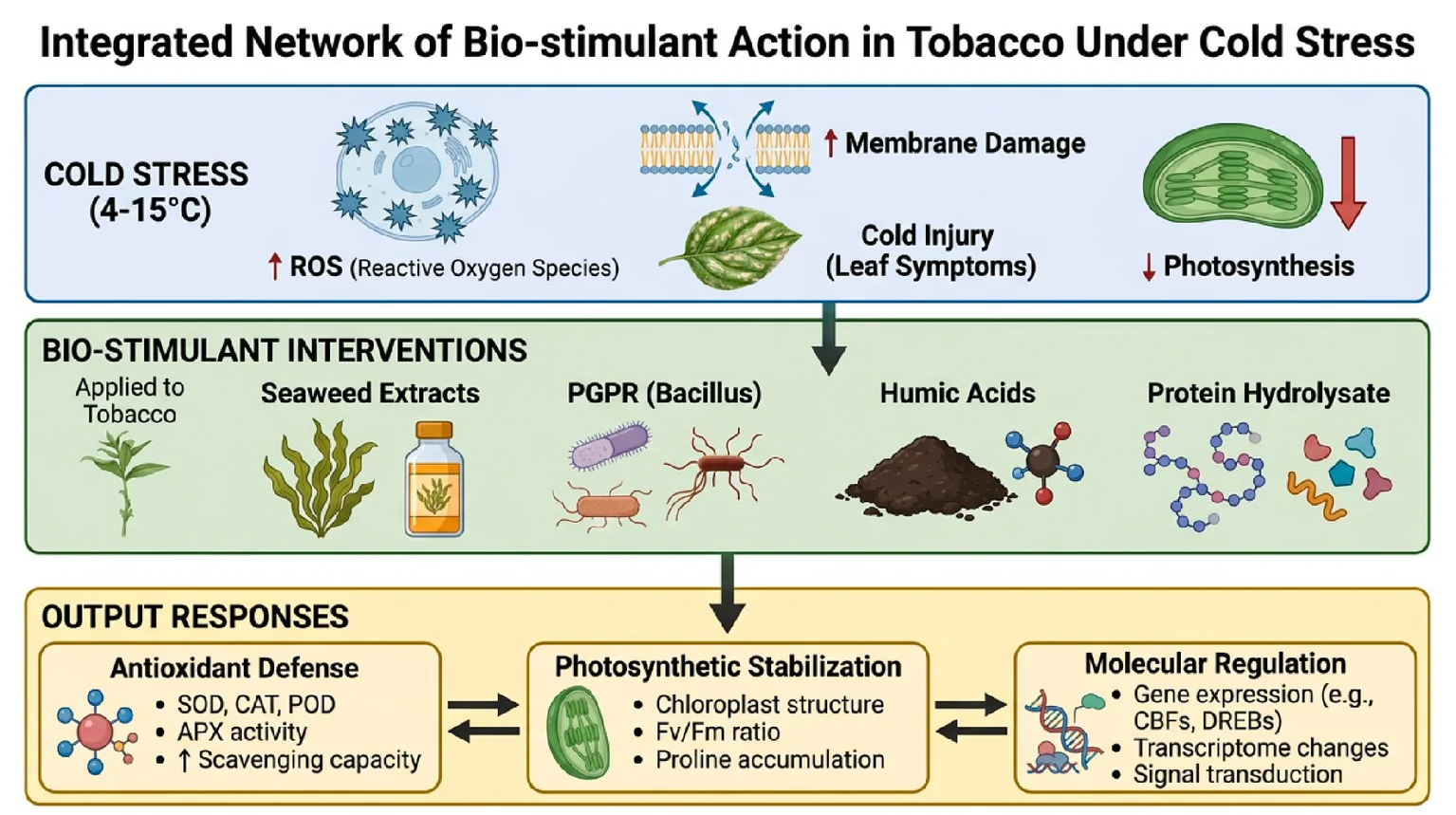
Overview of the physiological, biochemical, and molecular responses of tobacco plants to cold stress. Low-temperature exposure disrupts membrane stability, photosynthetic efficiency, water relations, and nutrient uptake, while promoting excessive accumulation of reactive oxygen species (ROS) and oxidative damage. Cold stress also activates calcium signaling, MAPK cascades, hormone-mediated pathways, and stress-responsive transcription factors, leading to osmotic adjustment, activation of antioxidant defenses, and metabolic reprogramming that collectively determine tobacco adaptation and survival under chilling conditions.

Tobacco (*Nicotiana tabacum* L.), a prominent member of the *Solanaceae* family, stands as one of the most economically and socially vital industrial crops worldwide (Gu et al., 2024). Originating in the Americas, its cultivation supports the livelihoods of millions, particularly in developing nations, while contributing significantly to national revenues through global trade. It is widely cultivated in temperate and subtropical areas, such as China, India, the United States, Brazil, and some African countries. Beyond its commercial utility defined by its nicotine content and alkaloid diversity, tobacco is a cornerstone of modern scientific research (Tsaliki et al., 2023). As a model plant, tobacco has also played a significant role in scientific research as an exemplary system for molecular biology, plant physiology, and genetic engineering, owing to its simple transformation process and well-defined genome (see Figure 1).

Abiotic stresses such as drought, salinity, temperature extremes, and heavy metals significantly impede plant growth and agricultural productivity (6). Among these, cold stress (CS) is especially harmful because it limits enzymatic and metabolic functions and causes oxidative damage by disrupting the redox balance within plant cells (7). The cultivation of tobacco is severely hampered by CS, which is defined as temperatures falling below 15 °C, especially during delicate growth stages (Li Yunnan et al., n.d.; Li et al., 2021). The CS damage is expressed in the disruption of the lipid bilayer structure of the plasma membrane. Cold stress disrupts cellular homeostasis by affecting the fluidity of the membrane, which leads to poor cell membrane integrity, increased extracellular electrolyte leakage, and diminished cellular compartmentalization (Manchanda et al., 2023). The expression of important cold-responsive genes, including those related to the C-repeat binding factor (CBF) pathway, is markedly affected, resulting in activation of downstream protective systems such as accumulation of osmolytes and antioxidant defense systems. Despite these adaptive responses, tobacco is very sensitive to CS, and its photosynthetic efficiency, chlorophyll content, and biomass accumulation are decreased (Su et al., 2025). Cold stress (CS) impairs enzymes and down-regulates genes related to light-harvesting complex proteins (LHCs), leading to increased photo-inhibition and reduced photosystem II efficiency. These disruptions directly diminish biomass growth and ultimately affect crop yield and quality (Rao and Zheng, 2025). CS also causes excessive reactive oxygen species production due to disturbances in the electron transport chain, prompting plants to activate antioxidant defenses and utilize non-enzymatic components to combat oxidative stress (Sachdev et al., 2021).

Bio-stimulants have been shown to enhance rhizosphere nutrient availability and microbial activity in various crops; however, tobacco-specific evidence regarding direct improvements in soil physical properties remains limited and requires further investigation (Rai et al., 2021; Johnson et al., 2024). According to the European Bio-stimulants Industry Council (EBIC), these substances enhance nutrient use efficiency, plant quality, and stress resilience, regardless of their nutritional content (Khalid et al., 2025). These bio-stimulants, microbial and non-microbial, each play a distinct role in enhancing plant adaptation to abiotic stress factors like cold stress in tobacco (Ahmed et al., 2023; Johnson et al., 2024). Owing to the growing demand for sustainable and climate-resilient agricultural practices, there has been a great interest in using bio-stimulants as a means by which tobacco production can be reduced in terms of stress (Nephali et al., 2020; Igiehon et al., 2025). As climate-resistant tobacco production becomes a more demanded commodity, there is still an urgent research gap (Igiehon et al., 2025). Despite the availability of positive effects of different bio-stimulants in individual studies, a mechanical perspective of the role that these compounds play in controlling antioxidant defense systems and preserving photosynthetic under cold stress in tobacco is yet to be achieved (Elumalai et al., 2025).

This review thoroughly examines the role of bio-stimulants in enhancing antioxidant defense and photosynthetic efficiency of tobacco under cold stress. It encompasses a comprehensive literature survey of the electronic databases with the selected keywords for Web of Science, Scopus, PubMed, Science Direct, and Google Scholar databases. It focused on peer-reviewed articles, reviews, and book chapters published between 2020 and 2025 where they were relevant to the study of bio-stimulants in response to cold stress; it also included earlier important publications that are well-explained how bio-stimulants work in response to cold stress. Repeated articles, studies involving languages other than English, and those with less than experimental integrity were eliminated from the assessment, besides those that were unrelated to research. The contents of literature were organized into thematic sections, such as classification and mechanisms of bio-stimulants, antioxidant defense systems, photosynthetic reactions, physiological and biochemical adaptations, as well as contemporary molecular and omics approaches to tolerance of chilling stress. The structured approach aims to integrate current knowledge, identify gaps in research, and suggest future research directions towards sustainable tobacco production under low-temperature conditions.

## 2 Classification and mechanisms of bio-stimulants

Bio-stimulants are naturally derived or microorganisms that can be used to promote growth, resilience, and productivity (Bhupenchandra et al., 2022). Many organic and inorganic complex combinations of useful microorganisms are used as bio-stimulants; they work through various mechanisms to enhance plant resistance, plant absorption, and promote physiological functioning (Mandal et al., 2023). Bio-stimulants are chemicals that stimulate plant growth and disease resistance but do not directly supply feed material (Drobek et al., 2019; Tavarini et al., 2018). Bio-stimulants, which are available in various forms to interact with environmental stressors (Kocira et al., 2018). This advancement enhances growth parameters and abiotic stress resistance, leading to greater productivity and quality products (du Jardin, 2015). Presently, it is challenging to categorize associated compounds and microbes comprehensively due to the absence of a legal or regulatory definition of plant bio-stimulants globally, including in the US and the EU. Conversely, researchers and authorities acknowledge beneficial bacteria and fungi as significant entities, whether they exist as free-living organisms or in rhizosphere or endosymbiosis associations.

One of the most researched and utilized bio-stimulants is seaweed extract, particularly that of *Ascophyllum nodosum*. Seaweed extracts have long been used in agriculture because of their high concentration of bioactive substances, such as polysaccharides, peptides, amino acids, vitamins, and minerals, which are thought to improve plant development and stress tolerance (Di Sario et al., 2025). These extracts’ methods of action include modulating plant metabolism, stimulating antioxidant defenses, and improving photosynthetic activity, particularly under unfavorable circumstances such as drought, low temperature, or salt. The main advantage of employing seaweed extracts is that they aid in promoting the production of plant growth regulators, such as auxins, cytokinins, and gibberellins, which are involved in cell division, root formation, and general plant growth (Mughunth et al., 2024). Moreover, the seaweed extracts prevent oxidative stress by inhibiting reactive oxygen species (ROS), thus alleviating the damage to cellular compounds such as proteins, lipids, and DNA. Research indicates that the use of seaweed extracts, especially of *Ascophyllum nodosum*, promotes the growth of roots, enhances the uptake of nutrients, and tolerance to biotic and abiotic stressors (Kumar et al., 2024). Together with the seaweed extracts, humic and fulvic acid, and organic matter, which is produced by the decomposition of plant and animal materials, are effective bio-stimulants (Liu et al., 2022).

**Figure 2:**
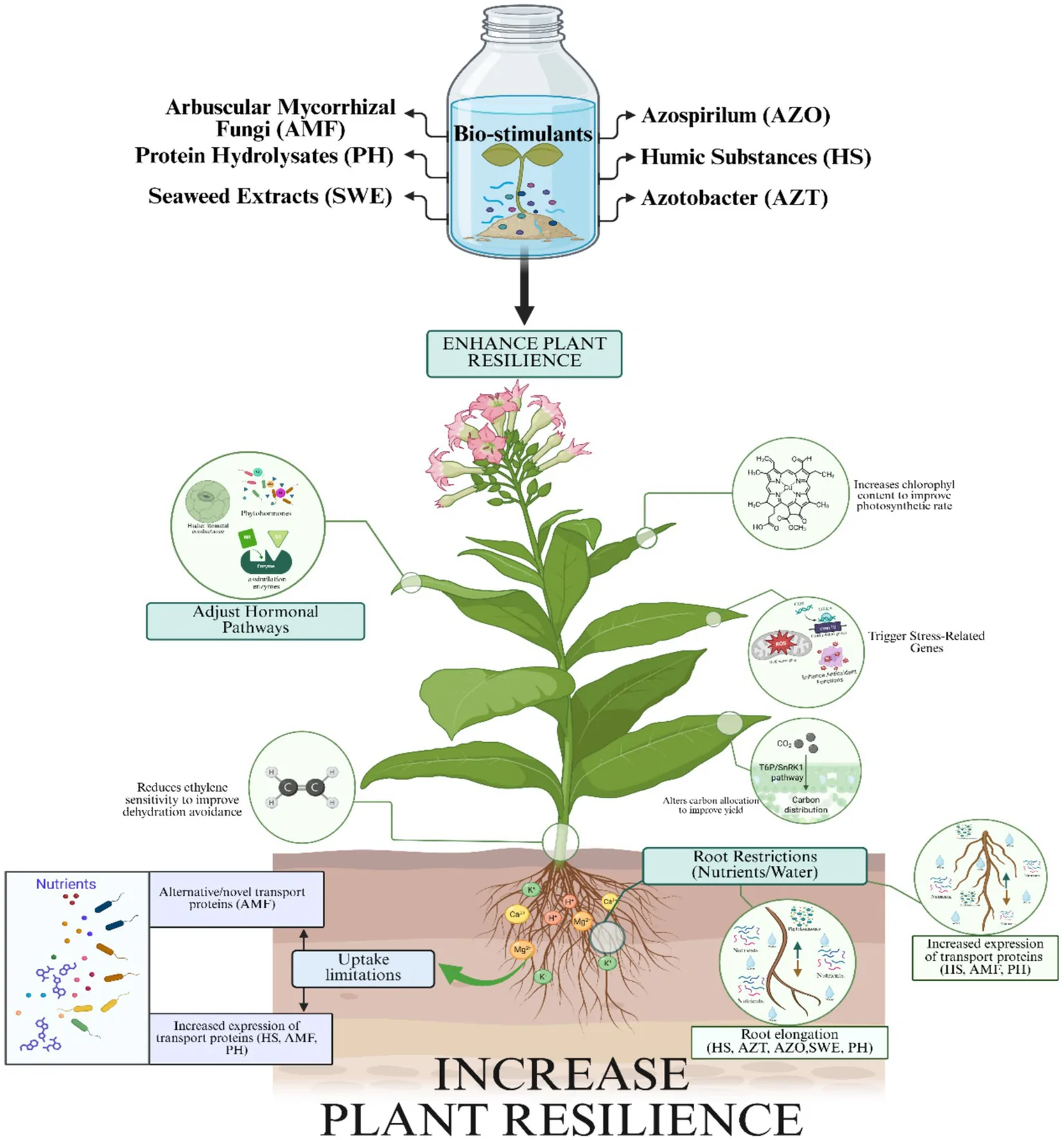
The mechanism of action used for bio-stimulant-induced increase in cold stress tolerance in tobacco. Microbial and non-microbial bio-stimulants are shown to enhance plant resistance capacity through stimulating antioxidant enzymes, regulating the signaling of phytohormones, accumulating osmolytes, triggering resistant gene expressions, and increasing nutrient uptake. These physiological and molecular integrated responses contribute to maintaining photosynthetic efficiency, less oxidative damage, better growth, and higher productivity in tobacco under cold stress.

Humic substances have been reported to enhance rhizosphere nutrient availability and microbial activity in various crops; however, tobacco-specific evidence for direct improvement of soil physical properties remains limited (Liu et al., 2021). It also improves the growth of a healthy root system and increases the uptake of nutrients. And enhance the activity of microorganisms in soil. As humic acids are less soluble, fulvic acids help chelate essential micronutrients and increase their bioavailability to plants (Shahrajabian and Sun, 2023; Figure 2). The application of bio-stimulants such as Arbuscular Mycorrhizal Fungi (AMF) and Humic Substances (HS), triggers a systemic response in tobacco, ranging from the modulation of ethylene sensitivity in the foliage to the enhancement of nutrient transport proteins in the root system. Bio-stimulants enhance antioxidant activity, stabilize membranes, and boost root conductivity, improving photosynthesis, nutrient absorption, and overall vigor under low temperatures (Singh et al., 2025). These acids enhance plant defenses by promoting phytoalexin production and activating resistance pathways, helping plants cops with environmental stress. Humic and fulvic acids have been demonstrated to promote plant growth in various stress situations, such as drought, salinity, and nutrient deficiency, by improving soil quality and facilitating nutrient absorption. However, according to Kumar et al. (2025), protein hydrolysates and amino acids, derived through enzymatic protein degradation, significantly enhance plant development and resilience against stress by providing essential nutrients for growth.

The amino acids, microbial inoculants, and minerals are necessary to support the physiological homeostasis of tobacco (*Nicotiana tabacum* L.) during unfavorable environmental conditions. Amino acids are extremely important not only as building blocks of proteins but also as signaling molecules that have an osmotic gradient and stabilize cellular membranes in cold and drought stress conditions (Trovato et al., 2021). Additionally, amino acids function as antioxidants by neutralizing free radicals and shielding plants from oxidative harm. Besides, protein hydrolysates and amino acids are antioxidants neutralizing the effects of free radicals and promoting the production of Heat Shock Proteins (HSPs) that help in preventing protein misfolding in tobacco leaves (Chakraborty and Newton, 2011; see Table 1).

**Table 1:** Mechanisms and effects of different bio-stimulants on photosynthesis under cold stress.

Bio-stimulant category	Mechanism of action	Effect on photosynthesis under cold stress	Reference
Seaweed extracts	Contain cytokinins, auxins, betaines; improve chloroplast stability and pigment content.	Increased chlorophyll retention, enhanced Fv/Fm, better gas exchange	Shaari et al. (2023)
Humic substances	Enhance nutrient uptake, stimulate root and microbial activity	Better water and nutrient availability, improved PSII efficiency	Chen et al. (2022)
Protein hydrolysates	Supply amino acids and peptides, improve stress enzyme activity	Stabilized electron transport, higher photosynthetic rate	Pasković et al. (2024)
Plant hormones	Exogenous cytokinins/ABA regulate stomatal aperture and stress signaling	Optimized CO₂ uptake, improved photochemical efficiency	Wang et al. (2022)
PGPR	Enhance nutrient uptake, produce stress-related phytohormones	Enhanced stomatal conductance, improved Fv/Fm, and increased chlorophyll	Anli et al. (2020)
Mycorrhizal fungi	Improve phosphorus uptake, water use efficiency	Increased PSII stability, sustained electron transport	Tang et al. (2022)
Amino acid formulations	Act as precursors for chlorophyll and osmoprotectants	Improved pigment biosynthesis and energy conversion	Quan et al. (2022)
Chitosan	Elicits defense responses, enhances chloroplast protection	Improved Fv/Fm and chlorophyll fluorescence under cold	Li et al., (2024b)
Fulvic acid	Improves metabolic activities, supports nutrient transport	Enhanced photosynthetic enzyme function and pigment stabilization	Yu et al. (2024); Song et al. (2025a)
Microalgae extracts	Source of vitamins, antioxidants, and polysaccharides	Protect the photosynthetic apparatus from oxidative stress	Vignaud et al. (2023)
Antioxidant-rich extracts	Provide non-enzymatic ROS scavengers (polyphenols, flavonoids)	Reduce oxidative damage to PSII, maintain chlorophyll content	Sayed et al. (2024)

Microbial inoculants like *Trichoderma* spp., Azospirillum spp., are an advanced method of improving the tobacco-soil relationship (Praveen et al., 2024). It has been noted that *Trichoderma* is able to help colonize tobacco roots, protecting against pathogens by secretion of volatile organic compounds (VOCs) that trigger systemic acquired resistance (SAR) across the plant (Tyśkiewicz et al., 2022). This colonization shows a considerable increase in the chlorophyll content and efficiency of the intrinsic antioxidant enzymes of the plant (Hassan et al., 2021). In the meantime, the nitrogen-fixing strain *Azospirillum* promotes the growth of tobacco not only by fixing nitrogen in the air but also by remodeling root structure by regulating local auxin levels to increase the overall root surface area to capture nutrients (Chen et al., 2024a). In addition to biological factors, bio-stimulants made of minerals, such as silicon (Si) and calcium (Ca_2_), are ineffective in making tobacco resistant to abiotic stress (Ali et al., 2024; Khalid et al., 2025). Silicon is sprayed onto the cell walls, which forms a physical barrier that improves mechanical strength and decreases transpiration rates with the regulation of stomatal conductance (Alayafi et al., 2022). It has also been discovered that the silicon-treated tobacco exhibits increased density of trichrome, thus leading to better ability to retain water and avoid biotic leaf-surface stressors, and also the oxidative damage caused by the UV. Furthermore, calcium is a key second messenger in tobacco signaling cascades; its influx activates calcium-dependent protein kinases (CDPKs), which mediate decoding cold-stress cues into physiological adaptive behaviors (Ravi et al., 2023). Bio-stimulants help plants adapt to abiotic stress through proper tree hormone fluctuations, efficient nutrient absorption, and activation of plant antioxidant stress mechanisms (Muhammad Aslam et al., 2022; Roychowdhury et al., 2025). They have a regulatory function of signaling pathways such as ABA, SA, and JA that are critical for stress recognition and stress response (Ma et al., 2022). The chemicals can improve the efficiency of photosynthesis and the overall resistance of the plant to cold stress by stimulating antioxidant enzyme activity, maintaining the structural integrity of cell membranes, and promoting chloroplast function under cold stress (Singh et al., 2022a; Singh et al., 2022b). Additionally, bio-stimulants facilitate nutrient uptake and support healthy plant growth under stressful conditions by improving soil properties and promoting root development. Humic substances improve soil by increasing porosity, water, and nutrient retention, enhancing root growth. Seaweed extracts and microbial inoculants support nutrient absorption, promoting plant development in difficult conditions (Nabi et al., 2025).

Microbial inoculants improve nutrient absorption, particularly nitrogen, vital for protein synthesis and plant growth, while also inducing stress-responsive gene expression in plants (Praveen et al., 2024). The expression of genes that react to stress is crucial for the survival of plants in difficult environmental situations. These genes code for proteins involved in stress tolerance mechanisms, such as antioxidants, osmoprotectants, heat shock proteins, and transcription factors that regulate the expression of other stress-related genes. Bio-stimulants can trigger the activation of these genes, helping plants manage various stresses such as drought, salinity, cold temperatures, and nutrient deficiency (Huang et al., 2021). Inoculation with *Trichoderma* activates genes for stress tolerance, including heat shock proteins and antioxidant enzyme coding (Geng et al., 2025). Seaweed extracts enhance drought and salt tolerance in plants by activating stress-responsive genes, including antioxidant enzyme genes like SOD and CAT, enabling protective mechanisms that aid survival under adverse environmental conditions (Elumalai et al., 2025).

Additionally, bio-stimulants are recognized for enhancing antioxidant enzyme activities, which are vital components of plant defense during stress (Mansoor et al., 2022). Excessive reactive oxygen species (ROS) harm cellular components (Rakkammal et al., 2022). Enzymes are vital during abiotic stress, as oxidative stress increases. Bio-stimulants increase the function of antioxidant enzymes as well as initiate defense mechanisms such as the production of compatible solutes, an aid in maintaining cell integrity and osmolar balance in the event of cellular stress (Cannea and Padiglia, 2025). Bio-stimulants enhance antioxidant enzyme action and defensive mechanisms in plants, including the production of protective solutes for cell integrity and osmolar balance (Abdel Latef et al., 2026). They involve agents like arbuscular mycorrhizal fungi, phytohormones, and various acids, which improve stress resistance by modifying hormonal pathways and upregulating stress response genes. These substances also promote carbon uptake, root growth, and chlorophyll increase, contributing to improved resilience and productivity in adverse environmental conditions.

Antioxidant enzymes protect plants from reactive oxygen species (ROS) produced during stress. Bioactive compounds, like amino acids and seaweed extracts, enhance these enzymes, thus increasing plant resilience against oxidative stress (Kumar et al., 2023). Research shows that these compounds boost antioxidant levels, particularly in tobacco, improving enzyme activity and mitigating cold stress effects (Berthon et al., 2021). In stressed tobacco plants, *Ascophyllum nodosum* extract enhances SOD activity. Amino acids from degraded proteins boost glutathione synthesis, which protects against oxidative damage and supports antioxidant functions by neutralizing ROS (Valgimigli, 2023). Additionally, proline and glycine betaine enhance plant resistance to oxidative stress by stabilizing cell membranes and regulating osmotic balance (Obeme-Nmom et al., 2024). Bio-stimulants, particularly amino acids, also elevate the expression of antioxidant-related genes, thereby improving stress-responsive proteins for redox equilibrium and ROS scavenging (Hasanuzzaman et al., 2021).

### 2.1 Influence of bio-stimulants on photosynthetic efficiency

**Figure 3:**
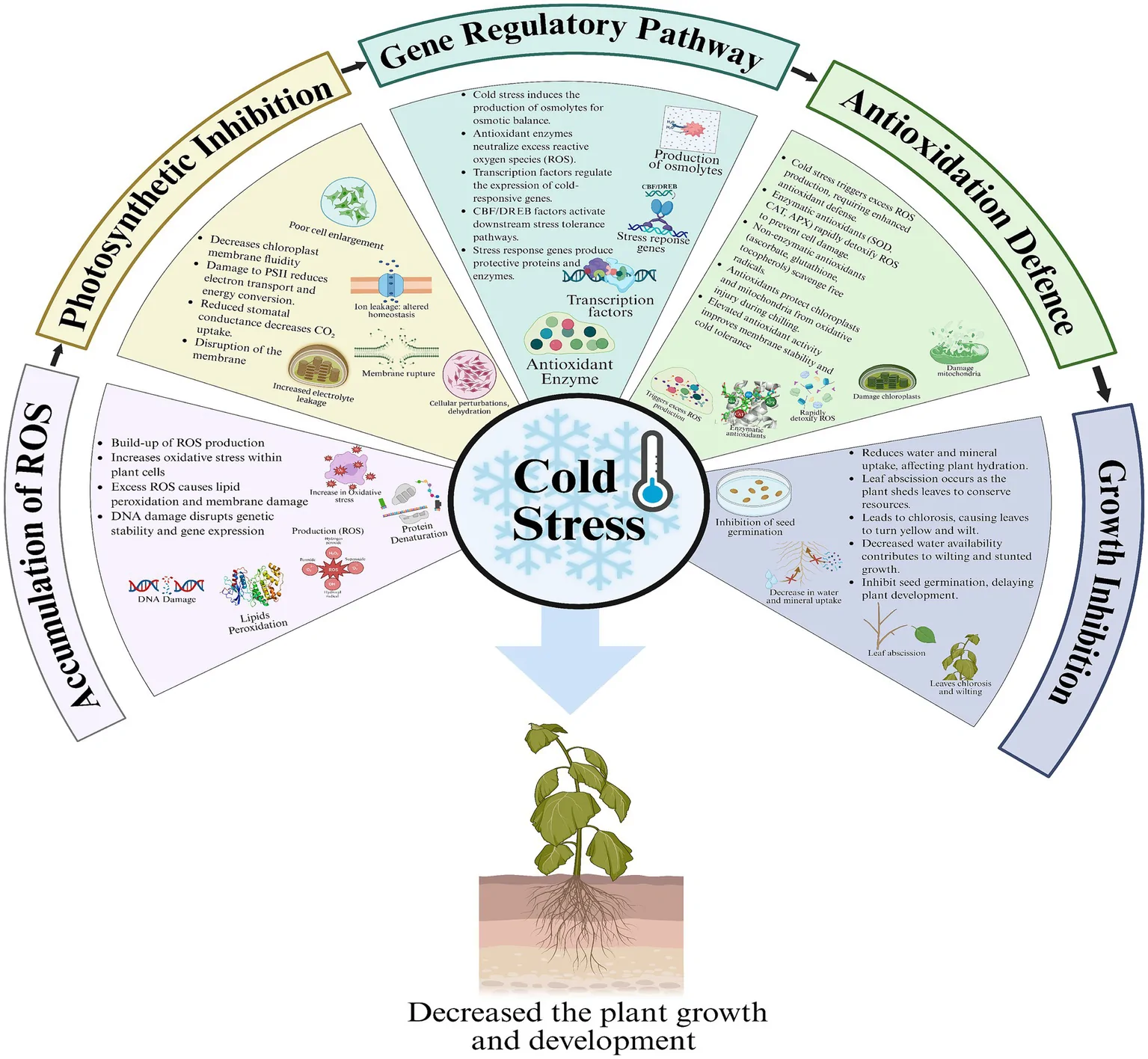
Cold stress affects plant growth by inducing photosynthetic inhibition, accumulation of ROS, suppression of growth, and physiological imbalances, while also triggering gene regulatory and antioxidative pathways that enhance stress tolerance.

Bio-stimulants significantly enhance tobacco photosynthesis, particularly under challenging conditions like low temperatures (Rajesaheb et al., 2025). CS damages chloroplasts, disrupts light harvesting, and inhibits carbon assimilation enzymes in plants (Ansabayeva et al., 2025). Effective photosynthesis under cold stress is crucial for growth, productivity, and plant health (Sharma et al., 2019). It significantly mitigates cold stress effects on photosynthesis by enhancing photosynthetic mechanisms. It helps tobacco plants retain chlorophyll and carotenoid levels during cold stress. Chlorophylls are essential for capturing light energy in photosynthesis, while carotenoids protect chlorophyll from reactive oxygen species (Liu et al., 2022). CS reduces chlorophyll content and photosynthetic activity, impairing the plant’s ability to utilize light effectively (Li et al., 2024a). However, the treatment with bio-stimulants boosts carotenoid production, offering improved photo-protection against oxidative stress (Knfe Yakob et al., 2024). The major classes of bio-stimulants and their mechanisms of action involved in enhancing cold stress tolerance in tobacco are summarized in Figure 3. An essential factor of photosynthesis that bio-stimulants positively affect is the stabilization of photosystems (PSI and PSII) (Billah et al., 2024). PSI and PSII are key elements for the photosynthetic electron transport chain (Huang et al., 2020). Cold stress potential destabilizes these photosystems and thus disrupts the transportation of electrons in the system and reduces the synthesis of ATP and reduced triphosphate. Reduced synthase primarily affects the Calvin cycle and carbon fixation (Tang et al., 2023). In this context, the stabilization means that the plant reinforces its membrane, defends itself with antioxidants, and protects its proteins (Khan, 2025).

Besides stabilizing the photosynthetic system, bio-stimulants increase Fv/Fm and electron transport rate (ETR), also as indicators for photosynthesis performance. The Fv/Fm ratio expresses the ability of photosystem II to utilize light energy and is most often used as a gauge of how healthy and efficient the photosynthetic system is. In general, if exposed to cold stress, the Fv/Fm ratio decreases when photosynthesis and the photochemical quenching of PSII are inhibited (Lysenko et al., 2022). Thus, stimulated relief in the measured time course of photon uptake and increasing the relative efficiency of electron transport in the photosystem as a component of a more rapid, bio-stimulants triggered photon relaxation was noted (Ali et al., 2022). Overall biochemical efficiency in photosynthetic systems is determined by the electron transport rate (ETR) (Lu et al., 2025). Bio-stimulants had a positive effect on the photosynthesis of tobacco plants under cold exposure by promoting carbon assimilation and accumulation of sugars (Singhal et al., 2022). Bio-stimulants have been found to increase the photosynthetic efficiency of tobacco under cold stress by increasing carbon assimilation and sugar accumulation, which are important factors to determine growth (Singhal et al., 2023). Cold conditions usually inhibit the Calvin cycle, resulting in less fixation of carbon, but application of bio-stimulants activates the enzymes in the Calvin cycle and enhances the activity of Rubisco, which, in addition to subsequently increased carbon fixation, also enhances sugar accumulation, which serves as an osmolyte and a reservoir for energy. Treated specimens exhibit high levels of soluble sugars, glucose, and sucrose, enhancing osmotic equilibrium and protecting the cell structure from the impact of low temperature (Rasheed et al., 2023). The inclusion of sugar concentration makes it possible to grow the plants as well by providing enough energy for metabolic functions. Bio-stimulants positively affect photosynthetic performance by regulating the homeostasis of the cellular energy, and thus allow the plants to optimally allocate resources for energy even under stress. It is essential for efficient photosynthesis that the overall structure of the photosynthetic apparatus is preserved, and if individual subcellular processes are a case in point, bio-stimulants regulate functional chloroplast protein stability and functionality independent of stress (Zhao et al., 2023). As climate change intensifies environmental stresses, bio-stimulants emerge as a sustainable solution for boosting crop productivity and resilience, necessitating further research to optimize their application.

### 2.2 Root microbiome modulation as a mechanism of bio-stimulant action under cold stress

The rhizosphere microbiome, comprising diverse bacteria, fungi, and actinomycetes, and other soil microorganisms living around plant roots, is essential for controlling plant growth, nutrient acquisition, and resistance to abiotic stressors (Pang et al., 2026). The subterranean microbial community is an extension of the plant’s physiological system in terms of the structure of the roots, hormone interactions, and metabolic balances (Al-Turki et al., 2023). The rhizosphere microbiome composition and function under cold stress conditions (between 4 and 15 °C) are significantly altered (Kannan et al., 2026). This lowered temperature stress decreases the enzymatic activity of microorganisms, affects nutrient cycling processes, and alters the structure of microbial communities, often shifting toward less beneficial, stress-tolerant, but functionally suboptimal microbial communities (Iqbal, 2026). The difference has a negative effect on the plant-microbe relationships and on plant resistance. An interesting strategy that has emerged to counteract these negative effects is the use of bio-stimulants, which selectively disrupt the root microbiome (Bakermans and Skidmore, 2011). Microbial inoculants, humic substances, and seaweed extracts have a specific ability to increase the amount and activity levels of soil microorganisms that positively support plants and suppress unwanted species (Li et al., 2026c). The changes increase the diversity of root exudates, draw in microbial communities, and improve rhizosphere signaling networks, thus restoring microbial functional balance (Li et al., 2026c). Therefore, bio-stimulants can improve the efficiency of nutrient uptake by plants, offer protection against stress, and modulate the expression of stress-responsive genes, particularly under cold stress (Punjamgod et al., 2026).

#### 2.2.1 Cold-induced dysbiosis of the rhizosphere microbiome

Low temperature stress (4–15 °C) profoundly changes the functions of microorganisms, their metabolism, and population structure in the soil plant feeding medium, and affects the structure and functional stability of the rhizosphere microbiome (Kibria et al., 2026). Physiological barriers impede microbial turnover and reduce soil-root interactions and functions, which, in turn, affect plant-associated microbial processes (Maura et al., 2026). In tobacco and related Solanaceae plants, cold conditions consistently lead to changes in microbial community composition, marked by a decrease in beneficial groups such as Proteobacteria and Actinobacteria, which are essential for nutrient cycling, organic matter breakdown, and plant growth (Liang et al., 2026). The decrease is correlated with a relative increase of cold-adapted or opportunistic microorganisms that oftentimes do not exhibit the mealybug growth-promoting effect in this instance (Zhang et al., 2026). One of the main results of this change is the inhibition of the root-colonizing rhizobacteria required for plant growth (PGPR) like *Pseudomonas fluorescens*, *Bacillus subtilis*, and *Azospirillum brasilense* (Maura et al., 2026). These microorganisms play a role in the production of auxins, mobilization of phosphorus, nitrogen conversion, as well as inhibit soil borne pathogens (Zhang et al., 2026). They are less abundant in cold environments, which reduces the ability of root-microbe interactions and leads to rhizoplane colonization by not-so-beneficial microbes (Hajji-Hedfi et al., 2026). In addition, cold stress negatively affects the production of many other microbial metabolites, such as trehalose and glycine betaine, which are important for stress alleviation (De Pessemier et al., 2026). Combined, these changes compromise induced systemic resistance (ISR) response and plant physiological and biochemical defenses of chilling stress.

#### 2.2.2 Bio-stimulant-mediated restoration of beneficial microbiome taxa

Bio-stimulants play an important role in recolonizing the microbial community of the rhizosphere ecosystem that is disturbed during chilling stress, which takes place when beneficial microbes are selectively stimulated and their functional activity is enhanced (Liu et al., 2026a). Humic acids in soil or nutrient solutions create positive physiological and chemical interactions with the growth of some bacterial families related to stress adaptation (de Oliveira Sátiro et al., 2026). Humic acid increased the relative abundance of Flavobacterium and Arthrobacter in tomato (*Solanum lycopersicum*) planted in chilling conditions (10 °C/day and 4 °C/night) by approximately 40–60% than the inoculated plants (Bayat et al., 2021). These bacteria are associated with the production of cold active enzymes (proteases and lipases) and extracellular polysaccharides that aid in soil aggregation, nutrient availability, and protection of roots from freezing damage (Khan et al., 2026). Bioactive polysaccharides, such as laminar in and alginates, which provide readily available carbon sources for rhizosphere microbes, promote the recovery of microbes from extracts of *Ascophyllum nodosum* (Syaifudin et al., 2026; Varela et al., 2026). The extraction and bio inoculation of these extracts into both soil and foliage of cattle pea under cold stress conditions (8 °C for 7 days) led to an increase of root associated populations of Bacillus and Pseudomonas species by two to threefold and increased enzyme activity (peroxidase) in the roots, while electrolyte leakage was reduced (Hilles et al., 2026). Remarkably, the inoculation tests revealed that *Bacillus subtilis* extracted from plants with the bio-stimulant seaweed had a significant positive effect on chilling tolerance of microbial untreated plants, providing direct evidence for the importance of changes in the microbiome when bio-stimulants like seaweed are used (Turan et al., 2026).

#### 2.2.3 PGPR-induced changes in rhizosphere community structure under chilling

Under low temperature conditions, plant growth-promoting rhizobacteria (PGPR) are active agents that alter the dynamics of the rhizosphere microbial communities via changes in microbial interactions, nutrient dynamics, and stress-related functions (Yang et al., 2026). Among those, *Pseudomonas fluorescens* strain Pf-5 is well known to produce an antimicrobial agent such as 2,4-diacetylphloroglucinol (DAPG), which is capable of solubilizing cold-sensitive fungal pathogens while being beneficial for the other bacteria (Nicotra et al., 2026). Under chilly conditions (12/4 °C day/night temperature), the use of Pf-5 highly enhances the diversity of the rhizosphere bacterial flora and enriches the numbers of genera involved in nitrogen transformation, such as *Nitrosospira* spp., and genera producing phytohormones such as *Rhizobium* spp. (Satognon and Akuku, 2026). The community changes increase the nutrient availability and promote root development under cold temperature stress (Choudhary et al., 2026). In contrast, metagenomic analyses reveal that the predominant rhizosphere microbiome (native rhizosphere) upon GB03 inoculation consists of genes for cold-shock proteins (CSPs), antifreeze proteins, antioxidant enzymes, and reactive oxygen species (ROS) detoxification systems begins to appear within 48 h after application and can persist for 10 °C exposure for extended periods (Elsayed et al., 2026; Hassan et al., 2026). This community-wide functional reprogramming enhances effective PGPR functions not just as promoters of plant growth, but also as effective regulators of plant uptake and acquisition of nutrients, rhizosphere stability, and increased plant resistance to cool conditions (Sharma and Pandey, 2026).

## 3 Integrating antioxidant defense and bio-stimulants to enhance cold stress in tobacco

**Figure 4:**
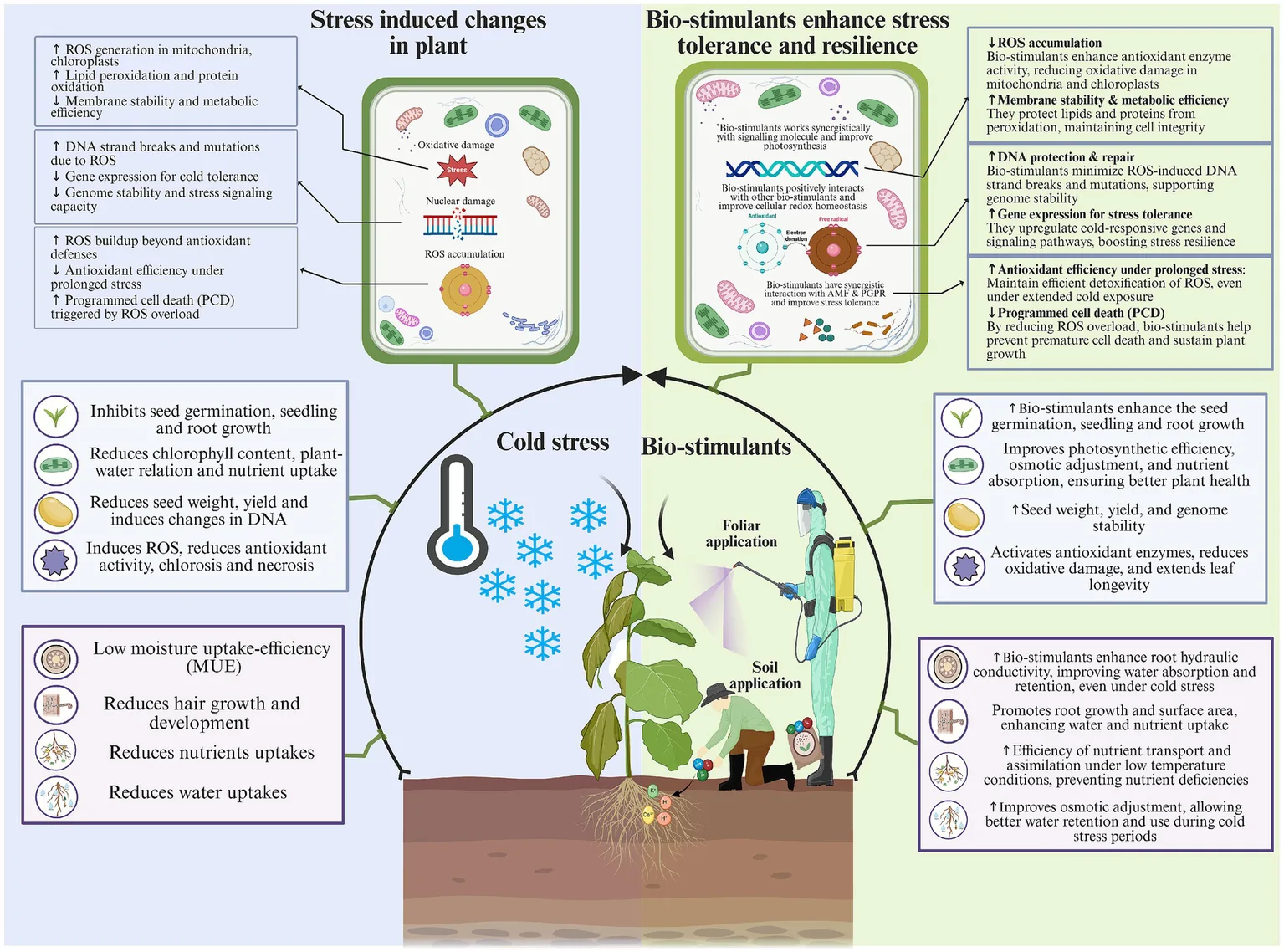
Bio-stimulants enhance cold stress resilience in plants through antioxidative defense, nutrient uptake, and stress-responsive gene regulation.

The antioxidant defense system has become a major center of attention in the study of plant stress physiology, as there is an immediate necessity for climate-resistant tobacco production. Sub-optimal temperatures negatively affect the photosynthetic productivity, respiration rate, and membrane integrity of tobacco, mainly by over-producing Reactive Oxygen Species (ROS), including hydrogen peroxide (H₂O₂) and superoxide anions (O₂^−^) (Nawaz et al., 2021; Phan and Schläppi, 2021). These ROS not only exhibit damaging properties but are also dual-faceted signaling molecules that activate programmed cell death (PCD) in tobacco leaf mesophyll cells once they reach threshold concentrations. To counter this, tobacco has developed a complex antioxidative system with enzymatic components—Superoxide dismutase (SOD), Catalase (CAT), Peroxidases (POD), and the enzymes of Ascorbate-Glutathione (AsA-GSH) system (APX, GR, MDHAR) and non-enzymatic compounds such as carotenoids, flavonoids, and tocopherols (Ighodaro and Akinloye, 2018). APX is especially important because it uses AsA to achieve redox homeostasis, and GR replenishes the reduced GSH pool needed to detoxify cells (Hasanuzzaman et al., 2020). Recent proteomic analyses of tobacco have shown that the localized chloroplast SOD and APX isoforms are strongly influenced by cold, implying that the redox poise in the chloroplast is the key determinant of tobacco cold-tolerance. Cold-tolerant varieties possess greater basal enzyme activity and lesser ROS build-up than cold-sensitive varieties (Sharma et al., 2016). Interventions from outside sources have demonstrated efficiency in enhancing this innate mechanism. The use of phytohormones such as Salicylic Acid (SA), *Brassinosteroids* (BR), and jasmonic acid (JA) is found to positively influence the expression levels of antioxidant-associated genes, including Cu/Zn-SOD and APX1, in *Nicotiana tabacum* by minimizing lipid peroxidation and ensuring that the process of photosynthesis is preserved (Soumare et al., 2021; Singh et al., 2022a). Moreover, certain transcription factors like CBF/DREB, NAC, WRKY, and MYB are responsible for controlling the induction of these networks associated with antioxidants through their responses to cold temperatures (Abdullah et al., 2022). The recent works have investigated the role of the non-coding RNAs, especially microRNAs (miRNAs), in the antioxidant response regulation during cold stress, and provide new insights into the post-transcriptional regulation (Kallugudi et al., 2025). The integrated physiological and molecular responses of tobacco to bio-stimulant application under cold stress are illustrated in Figure 4. In plants, miR398 is a highly conserved microRNA known to regulate the expression of copper/zinc superoxide dismutase (CSD) genes, which play key roles in scavenging reactive oxygen species (ROS) under stress conditions (Asim et al., 2026). In cold stress, ROS expression is elevated, and changes to miR398 level are mainly down-regulated, leading to increased expression of CSD1/CSD2 for protection against ROS; this represents the production of weaker antioxidant protection (Waseem et al., 2026). Enhanced ROS signaling due to bio-stimulants has been found to bypass in triggering miRNA regulatory networks, leading to a decreased miR398 expression, which, in turn, leads to an increase in the expression of CSD genes (Padmavathi et al., 2023). This results in improved detoxification of ROS and cold-tolerance by plant model studies (e.g., Arabidopsis and tobacco). Therefore, the actions of bio-stimulants are indirect on miR398 as they boost stress signals and allow for better hormonal interaction, leading to a higher antioxidant potential (Rai et al., 2021) In plants, miR398 is a highly conserved microRNA known to regulate the expression of copper/zinc superoxide dismutase (CSD) genes, which play key roles in scavenging Reactive Oxygen Species (ROS) under stress conditions (Asim et al., 2026). In cold stress, ROS expression is elevated, and changes to miR398 level are mainly down-regulated, leading to increased expression of CSD1/CSD2 for protection against ROS; this represents the production of weaker antioxidant protection (Waseem et al., 2026). Enhanced ROS signaling due to bio-stimulants has been found to bypass in triggering miRNA regulatory networks, leading to a decreased miR398 expression, which, in turn, leads to an increase in the expression of CSD genes (Padmavathi et al., 2023). This results in improved detoxification of ROS and cold-tolerance by plant model studies (e.g., Arabidopsis and tobacco). Therefore, the actions of bio-stimulants are indirect on miR398 as they boost stress signals and allow for better hormonal interaction, leading to a higher antioxidant potential (Rai et al., 2021) Their antioxidant defense mechanisms enhance photosynthetic efficiency, chlorophyll concentration, and membrane stability, which are vital for growth and productivity, especially in low-temperature conditions. Table 2 below displays the major enzymatic and non-enzymatic variables associated with cold stress tolerance, along with their activities and recent references.

**Table 2:** Role of antioxidant defense systems in tobacco under cold stress.

Category	Antioxidant component	Function in cold stress tolerance	Reference
Enzymatic antioxidants	Superoxide dismutase (SOD)	Converts superoxide radicals to H₂O₂; first line of defense	Obeme-Nmom et al. (2024)
	Catalase (CAT)	Breaks down H₂O₂ into water and oxygen	Sachdev et al. (2021a)
	Ascorbate peroxidase (APX)	Uses AsA to detoxify H₂O₂ via the ascorbate-glutathione cycle	ALKahtani et al. (2020)
	Glutathione reductase (GR)	Regenerates GSH from GSSG; maintains redox homeostasis	Mishra et al. (2023)
	Peroxidase (POD)	Detoxifies H₂O₂ using phenolic substrates	Wang et al. (2021)
	Monodehydroascorbate reductase (MDHAR)	Regenerates AsA from MDHA using NADPH	Li et al. (2024c)
Non-enzymatic antioxidants	Ascorbic acid (vitamin C)	Scavenges ROS directly; cofactor in APX reaction	(Kumar, 2024)
	Glutathione (GSH)	Maintains redox balance; substrate for GR and APX	Bela et al. (2022)
	Carotenoids	Quench singlet oxygen; protect chloroplast membranes	Zhang et al. (2021)
	Tocopherols (vitamin E)	Prevent lipid peroxidation; protect cellular membranes	Rao et al. (2025a)
	Flavonoids and phenolics	Act as secondary antioxidants; modulate ROS and signaling	Ball et al. (2023)
miRNA	Regulates antioxidant gene expression (CSD1/CSD2) under ROS	Bio-stimulant-enhanced ROS and hormone signaling suppresses miR398 → CSD upregulation	Li et al. (2022a)
Transcriptional regulators	Cbf/dreb, wrky, nac, myb	Regulate expression of antioxidant genes during cold stress	Goswami et al. (2022)
	Mirnas (e.g., mir398, mir319)	Post-transcriptional regulation of antioxidant enzymes	Huang et al. (2021)
Genetic engineering	Overexpression of SOD, APX, and GR	Enhances enzymatic ROS detoxification, improves cold tolerance	Rahman et al. (2024)
Bio-stimulant treatment	Seaweed extracts, sa, ja	Induce antioxidant enzymes and enhance stress resilience	Baltazar et al. (2021)
Omics tools	Transcriptomics, proteomics	Identify cold-responsive genes and proteins involved in antioxidant defense.	John Martin et al. (2024)
Integrated response	Antioxidant + photosynthetic protection	Synergistic role in maintaining plant growth	Rao et al. (2025b)

## 4 Physiological and biochemical responses of tobacco to cold stress

### 4.1 Membrane integrity and oxidative damage

**Figure 5:**
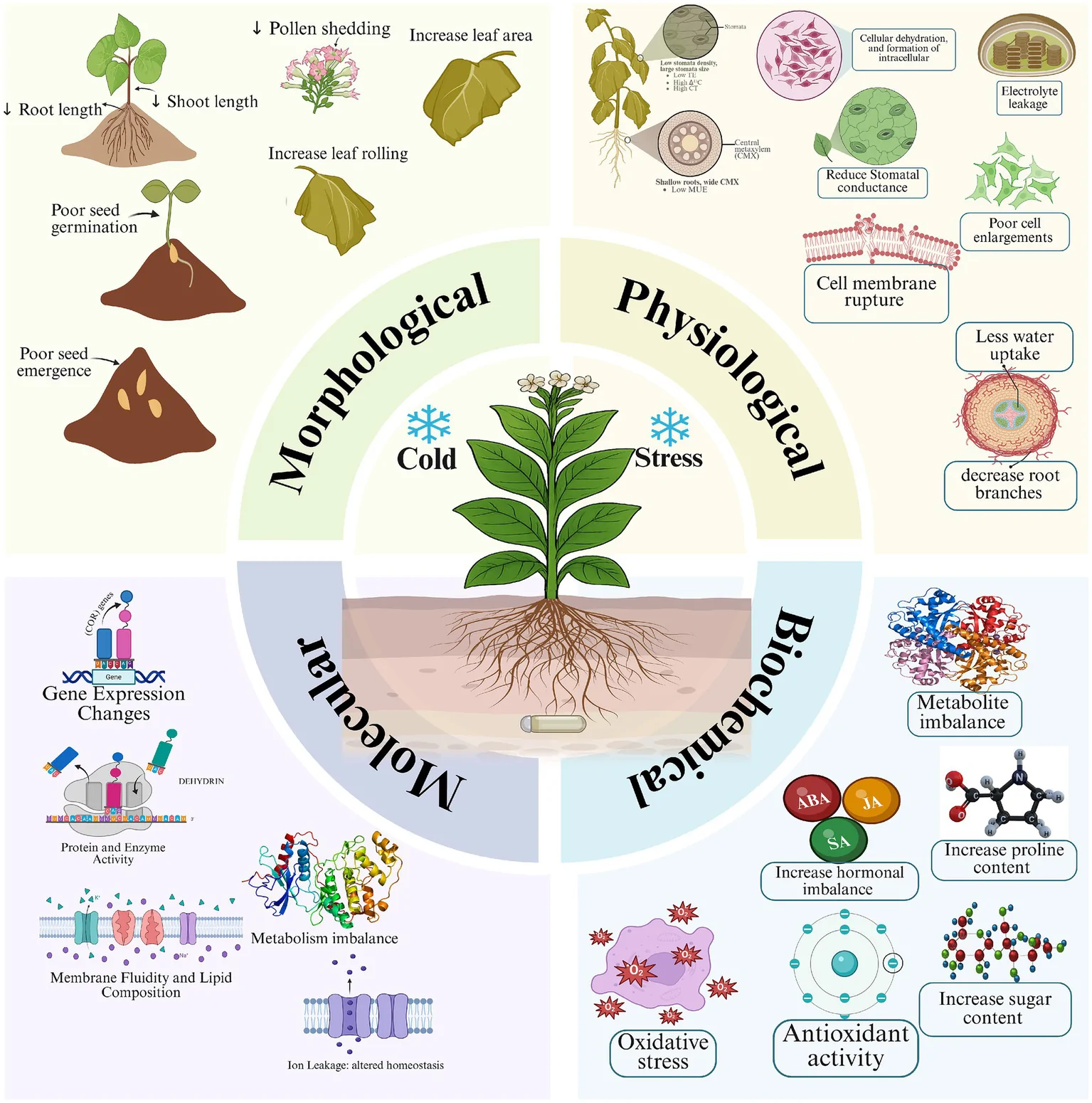
Cold stress adversely affects plants through morphological changes like poor germination, physiological alterations including dehydration and stomatal conductance reduction, biochemical imbalances with elevated proline and oxidative stress, and molecular disruptions.

Cold stress, which includes freezing stress at temperatures below 0 °C and chilling stress at temperatures between 0 °C and 15 °C, poses a serious threat to plant life and production, particularly for warm-adapted species like tobacco (Bhattacharya, 2022). Changes in gene regulatory systems, oxidative stress, and damage to photosynthesis are the main ways that cold stress affects a variety of physiological and biochemical processes in plants (Gu et al., 2024). One of the primary consequences of cold stress in plants is the rapid accumulation of ROS, such as superoxide anion radicals (O₂^−^), hydrogen peroxide (H₂O₂), and hydroxyl radicals (•OH) (Mir et al., 2024; Ahmad et al., 2025). ROS are secondary metabolites of abnormal photosynthesis in chloroplasts, mitochondria, and peroxisomes, which are crucial for respiration, energy homeostasis, and photosynthesis in plants (Rao et al., 2025). Under optimal conditions, a strong antioxidant defense system is available to plants to counteract these free radicals, including enzymes such as SOD, CAT, APX, and GR, and non-enzymatic antioxidants such as ascorbic acid, glutathione, and flavonoids (Berwal et al., 2020; Rajput et al., 2021). The oxidative stress, resulting from cold-induced enzyme deactivation and energy depletion, is extremely detrimental to the detoxifying mechanisms, resulting in significant oxidative damage (Vicidomini et al., 2024). Oxidative stress is also responsible for lipid peroxidation, resulting in the degradation of polyunsaturated fatty acids and the formation of malondialdehyde (MDA), a major indicator for measuring the extent of structural damage to the cell membrane and the resulting loss of cellular integrity (Wei et al., 2022; Figure 5). CS negatively impacts plants’ morphology, physiology, biochemistry, and molecular processes, causing poor seed germination, shortened root/shoot lengths, leaf curling, and reduced pollen release. In addition to oxidative stress, the photosynthetic apparatus of the tobacco plant is very sensitive to thermal fluctuations, which also affects the biosynthesis of alkaloids and secondary metabolites (Nazari et al., 2024). Cold stress causes a substantial reduction in photosynthetic efficiency due to the impairment of both light-harvesting complexes and carbon fixation in the broad leaves of tobacco (Wei et al., 2022). Cold stress also affects the thylakoid membrane, where reduced lipid fluidity causes physical destabilization of the Photosystem II complex, leading to reduced electron transport, as reflected by the marked reduction in the Fv/Fm ratio (Rosli et al., 2025). This photo-inhibitory effect not only reduces the production of vital chemical energy in the form of ATP and NADPH but also leads to the production of singlet oxygen (^1^O_2_), thereby accelerating the degradation of chlorophyll and the characteristic leaf chlorosis found in chilled tobacco seedlings (Pospíšil and Prasad, 2014). At the same time, the process of light-independent photosynthesis in the Calvin cycle is impaired due to lowered kinetic activity of the Rubisco enzyme and lowered availability of (CO_2_) due to stomatal closure induced by chilling stress (Dhatwalia et al., 2025; Nazari et al., 2024).

At a structural level, cold stress injury in tobacco is primarily manifested through bio-membrane instability in foliar and root tissues (Liu et al., 2022). The loss of kinetic energy within the lipid bilayers causes a phase transition from a liquid-crystalline to a rigid gel-like solid, which severely disrupts the cell membrane’s function as a selective barrier (Magalhães et al., 2024; Jambunathan, 2010). In addition to the leakage of electrolytes, the stiffening of the plasma membrane suppresses the activity of aquaporins, further suppressing the hydraulic conductivity of tobacco roots directly and exacerbating physiological drought with the presence of soil moisture (Mishra et al., 2023). This structural damage continues to damage the tobacco root system, which is inhibited in the development of the lateral root and root hair density, which, in turn, limits the uptake of nitrogen and potassium, which are critical to leaf development (Zhou et al., 2024b).

### 4.2 Hormonal signaling networks under cold stress

Cold stress activates a complex of tobacco signaling pathways with parameters such as calcium circuits, mitogen-activated protein kinases (MAPK), and reactive oxygen species (ROS) (Qian et al., 2024). ICE1 activates cold-responsive pathways in plants by binding to CBF/DREB1 gene promoters, leading to COR gene expression for cold acclimation. Its stability is regulated by modifications, and seaweed bio-stimulants may enhance its function (Hwarari et al., 2022). This signaling promotes cold-sensitive transcriptional programs, with the highest activity in the CBF (C-repeat binding factor)/DREB1 (dehydration-responsive element-binding) module (Li et al., 2020). CBF expression triggered by low temperatures binds to CRT/DRE motifs in Cold-Regulated (COR) gene promoters (Zhou et al., 2022). Recent studies on the NtCBF signaling pathway indicate that CBF proteins can also control the expression of cell wall remodeling enzymes, including xyloglucan endotransglucosylase/hydrolases (XTHs), which adjust cell wall elasticity in response to freezing-induced mechanical injury (Guo et al., 2018). The synthesis of osmolytes (proline, soluble sugars) and the production of dehydrins are downstream responses (Ritonga et al., 2021). Moreover, the transcription factor families, including MYB, WRKY, NAC, and bZIP family, are shown to act as downstream regulators of hormonal networks (Li et al., 2025). Hormonal regulation is a major factor in cold adaptation of tobacco. During low temperatures, abscisic acid (ABA) accumulates, which plays a role in stomatal closure and the expression of stress-related genes. ABA works in synergy with CBFs, and jasmonic acid (JA) and salicylic acid (SA) are important signaling molecules that generate antioxidant signatures and modify root structure (Chang et al., 2020).

### 4.3 Epigenetic regulation and stress memory

In addition to the complexity created through the use of epigenetic control mechanisms, histone acetylation and methylation regulate the ability to access stress-responsive genes. It has been found that histone deacetylases (HDACs) act as molecular switches in the case of tobacco plants, where they allow for the creation of a “hypothetical epigenetic memory” in cases of cold stress (Ramakrishnan et al., 2022). This memory allows for the expression of the genes due to the demethylation of the DNA in the promoter regions of NtCOR genes, which enables fast transcription when cold stress occurs. Such a phenomenon can be described as “stress priming” (Kim et al., 2020). Moreover, miRNA-319 and miRNA-398 help regulate the transcription factors and enzymes involved in antioxidation (Ijaz et al., 2024). Studies using metabolomics and proteomics revealed that there is an accumulation of compatible solutes, including trehalose, raffinose, and glycine betaine, in tobacco during cold stress in order to keep the osmolality constant (Kaur et al., 2024; Gulia et al., 2025). LEA proteins and heat shock proteins (HSPs) are regulated under cold stress to prevent protein aggregation (Jeyachandran et al., 2023). Omics techniques have greatly helped in understanding cold acclimation in Tobacco, providing possible means of intervention (Sakina et al., 2019). To survive, prompt initiation of signaling processes and effective protective measures should be provided, an approach known and accepted in plants’ stress responses biology, and is the basis for the application of to improve cold resistance (Ding et al., 2022). Table 3 summarizes the various physiological and biochemical alterations observed in tobacco under cold stress and their respective indicators.

**Table 3:** Key physiological and biochemical effects of cold stress in tobacco and their associated markers.

Category	Effect under cold stress	Indicators/markers	References
Cell cycle regulation	Arrest of cell cycle progression in meristematic tissues	Cyclins ↓ 30–50%, CDK expression ↓ 25–45%, mitotic index ↓ 35–60%	Qi and Zhang (2020)
Chloroplast integrity	Thylakoid swelling, photo damage, and disrupted grana structure	Chloroplast density ↓ 20–40%, thylakoid disorganization increased by 30–50%	Chen et al. (2014); Venzhik and Moshkov (2023)
Hormonal signaling	ABA, JA, SA, ET regulate cold response	ABA ↑ 2–5-fold, JA ↑ 30–70%, SA ↑ 20–60%, ethylene signaling genes ↑ 25–50%	Adhikari et al. (2022); Khan (2025)
Membrane integrity	Rigid membranes, increased electrolyte leakage	MDA ↑ 40–90%, ion leakage ↑ 30–80%, membrane stability index ↓ 20–45%	Rawat et al. (2021)
Metabolic shifts	Reprogramming of primary/secondary metabolism	Amino acids ↑ 20–60%, soluble sugars ↑ 30–80%, phenolics ↑ 25–70%	He et al. (2023); Song et al. (2025b)
Osmotic adjustment	Solute accumulation for osmotic balance	Proline ↑ 50–300%, soluble sugars ↑ 25–80%, glycine betaine ↑ 20–70%	Nawaz and Wang, (2020); Jabeen et al. (2022)
Oxidative stress	Excess ROS generation, lipid peroxidation, oxidative damage	H₂O₂ ↑ 40–100%, O₂^−^ ↑ 30–80%, MDA ↑ 50–120%, SOD ↓ 20–40%, CAT ↓ 15–35%, APX ↓ 20–45%	Qamer et al. (2021); Zhang et al. (2021)
Photosynthesis	Impaired PSII, reduced CO₂ fixation, photoinhibition	Fv/Fm ↓ 15–35%, chlorophyll a/b ↓ 20–40%, Rubisco activity ↓ 25–50%	Muhammad et al. (2021); Kılıç et al. (2023)
Respiration rate	ATP deficiency, inhibition of mitochondrial enzymes	Respiration rate ↓ 20–45%, ATP content ↓ 25–50%	Dong et al. (2020)
Root morphology	Reduced root growth, altered root architecture	Root length ↓ 20–50%, lateral roots ↓ 30–60%, root hairs ↓ 25–55%	Balliu et al. (2021); Karlova et al. (2021)
Signal transduction	Ca^2+^ signaling, MAPK cascade, ROS-dependent signaling	Cytosolic Ca^2+^ ↑ 2–4-fold, MAPK activity ↑ 40–100%, ROS-responsive genes ↑ 2–6-fold	Su et al. (2024)
Stomatal regulation	Closure of stomata, reduced gas exchange	Stomatal conductance ↓ 30–70%, CO₂ uptake ↓ 20–60%	Harrison et al. (2020)
Sugar metabolism	Cold-induced changes in sucrose/starch metabolism	Hexoses ↑ 25–70%, sucrose ↑ 20–60%, invertase activity ↑ 30–80%	Savitch et al. (2000)
Transcriptional regulation	Induction of CBF, COR, NAC, WRKY genes	CBF/DREB ↑ 3–10-fold, COR15A ↑ 5–15-fold, MYB ↑ 2–6-fold, WRKY ↑ 2–8-fold	Li et al. (2022b); Zhou et al. (2024a)
Water status	Water loss due to reduced uptake and increased transpiration	Relative water content ↓ 15–35%, ABA ↑ 2–5-fold, leaf water loss ↑ 20–50%	Cen et al. (2020)

## 5 Photosynthetic efficiency as an indicator of stress tolerance in tobacco plants

The studies conducted in recent years reveal that the use of bio-stimulants significantly increases photosynthetic effectiveness in cold stressed tobacco through health maintenance of leaf metabolism and increase of resistance to stress (Ma et al., 2026). Seaweed Extracts have been described as helping to preserve chlorophyll content and maintain photochemical processes by enhancing compatible solute biosynthesis and natural growth regulators (Saffari et al., 2026). Protein hydrolysates increase the efficiency of nitrogen use and amino acid metabolism, providing essential nitrogen components for the synthesis of protein and rapid recovery after chilling stress of photosynthetic tissues (Dlačić et al., 2026). Similarly, humic substances stimulate expression of genes linked to photosynthesis, helping in more efficient assimilation of carbon and extracellular tolerance to cold (Chen et al., 2024a). Recent studies have revealed that application of bio-stimulants can significantly improve tobacco photosynthetic efficiency under cold stress through the maintenance of proper metabolism of leaves and increased stress tolerance (Abdel Latef et al., 2026). Seaweed extracts have also been observed to preserve chlorophyll levels and to aid with photochemical processes, via an increased compatible solutes accumulation and natural growth regulator (Ali et al., 2021; Elumalai et al., 2025). Protein hydrolysates improve N uptake and amino acid metabolism, providing essential amino acid resources for protein synthesis and rapid repair of photosynthetic organs after chill stress (Balabanova et al., 2026). Similarly, humic substances promote photosynthesis related gene expression, promoting greater carbon uptake and cold tolerance (Ding et al., 2019).

Microbial bio-stimulants include plant growth-promoting rhizobacteria (PGPR), which increase the nutrient uptake and root function through increased levels of water and minerals, thus improving the photosynthetic efficiency (Chaffai et al., 2024). Moreover, they modulate phytohormones, as well as initiate stress responses, and promote enhanced performance of tobacco plants to chilling stress (Ullah et al., 2026). Research has demonstrated that treated plants retain more chlorophyll, have better gas exchange and higher biomass production than untreated plants (Rasulov et al., 2026). The incorporation of microbial bio-stimulants, especially plant growth-promoting rhizobacteria (PGPR), helps in assimilating nutrients and function of the root environments, thereby increasing the efficiency in the photosynthetic process through water and mineral availability (Li et al., 2026a). They also affect the regulation of phytohormones and the induction of stress response and preparing the tobacco plants to carry out better under chilling stress. Treated plants have been found to have more chlorophyll remaining, better gas exchange and higher plant growth than untreated plants (Ding et al., 2019).

## 6 Limitations and variable responses of bio-stimulants under cold stress

The use of bio-stimulants as a means to promote cold stress tolerance has been shown in many studies, however, there is some evidence that their response may not be consistent for every plant species, genotype and environmental condition (Yadav et al., 2026). The efficacy of bio-stimulants depends on their composition, application technique, dose and developmental stage, and the intensity of cold stress and exposure period. However, there have been limited reports on but inconsistent or even negative responses after bio-stimulant applications (Mousavi et al., 2026).

It is also essential that application be administered at the correct rate to ensure effectiveness. Stress adaptation usually happens in the presence of moderate levels of the seaweed extracts or humic substances or the protein hydrolysates, but an overabundance might cause more stress through hormonal imbalance, altered nutrient availability or higher metabolic expenditures, which can ultimately inhibit growth rather than reduce stress (Sana et al., 2026). Higher concentration of the treatment did not result in any increase in chlorophyll content, increase in biomass and activities of the antioxidant enzymes compared to untreated, which means that there is an optimum dosage for increasing white encompassing optimal responses (Sharma and Malaviya, 2026).

The environment is also variable, resulting in variable outcomes. It may be pointed out that at moderate chilling temperatures, plants are already able to withstand the stress due to chilling without any external factors, which reduces the actual benefits of using bio-stimulants (Kamanga et al., 2026). However, at very cold and prolonged stressful conditions, negative effects associated with bio-stimulants could not be overcome, and photosynthesis could be impaired (Devireddy et al., 2021). Further, results from different experiments may be hard to repeat due to the variability of the soils, microbiological populations, nutrient levels, and agricultural practices involved (Le et al., 2026). Another limitation is that there are no uniform formulations or experimental techniques. It is very difficult to compare different commercial bio-stimulant products and to link the variety of chemical compositions, bioactive components, and microbial communities used in these products to the reported benefits (Bhupenchandra et al., 2022). Furthermore, many studies are conducted in greenhouse conditions where conditions are explicitly controlled and only a few experiments have been performed over a long period in different environmental conditions to evaluate their long-term effectiveness (Zahid et al., 2026). Existing evidence, therefore, has a mixed message on how tobacco reacts to bio-stimulants, implying that this approach may be beneficial in improving tobacco’s resilience to cold stress under some circumstances, but under other conditions (Li et al., 2026b). Moreover, no economic evaluation has been published on the cost-effectiveness of applying bio-stimulants in tobacco cultivation under cold stress conditions. The return on investment (ROI), labor costs and cost effectiveness for smallholder farmers is yet to be analyzed. Moreover, research is needed on multi-location field tests, uniform application procedures, dose–response experiments, and multi-omics results, to identify the specifics when bio-stimulants can be seen to improve cold tolerance and when they cannot (Alrasheed et al., 2026; Wang et al., 2026).

## 7 Comparison of bio-stimulants with conventional cold stress mitigation strategies

One of the major approaches for increasing the cold tolerance has relied on the use of chemical priming agents, as well as manipulating plant growth regulators exogenously, e.g., abscisic acid (ABA), salicylic acid (SA), and jasmonic acid (JA) (Ali et al., 2026). These treatments are successful in eliciting a signaling pathway that responds to stress and in helping cells to adjust osmotically and in increasing antioxidant enzyme activity, protecting cells from damage during chilling stress (Chen et al., 2026). However, the effects of these often are short-lived and are greatly affected by the time, concentration, and environmental factors of application. An excess amount of hormones can lead to disruption of the natural hormonal balance that will adversely affect plant growth and development (Wang et al., 2024). In comparison, bio-stimulants seem to be a more holistic and complex solution, as they affect nutrient uptake, microbial relationships, hormonal control, antioxidant protection, and metabolic adjustment (Azzam et al., 2026).

Bio-stimulants activate several stress response pathways rather than a single signaling pathway. Consequently, enhancing the cold tolerance of the plants increases soil health, soil nutrient use efficiency, root growth, and overall yield (Seberi Riseh et al., 2026). An additional significant difference is sustainability. Chemicals and synthetic hormones generally require more than one application, which increases production costs or causes environmental problems due to excessive application (Ateeq et al., 2026). However, many bio-stimulators are of natural or renewable origin and stimulate beneficial microorganisms in the soil, which makes them more in line with sustainable farming practices. Bio-stimulants are not all equally effective, however, as their activity depends on their genotype, environment, formulation composition, and application technique (Liu et al., 2026b). These methods should not be seen as competing technologies, but future research should work on combining them. Level doses of hormone (LD) plus hormonal (or non-hormonal) bio-stimulants by microbes or other means may have synergic effects as they stimulate stress signals and promote better assimilation of nutrients, and increase physiological resilience (Khurshid et al., 2026). Field tests of the effectiveness of the treatments, ABA, SA, JA, seaweed extracts, and microbial bio-stimulants under the same cold stress conditions would provide significant information for establishing more efficient techniques for producing climate-smart tobacco.

## 8 Molecular insights and omics approaches

The bio-stimulants (Bs) have also proven to be very effective in alleviating abiotic stress that causes CS through activating essential pathways in enhancing plant stress resilience. With agriculture increasingly facing unprecedented challenges caused by changes in climate, a clear understanding of the molecular mechanisms underlying the action of bio-stimulants is needed to develop highly resistant crops (Han et al., 2024). Current research utilizing omics technologies, including transcriptomic, proteomics, and metabolomics, has gained a great deal of information on the underlying molecular mechanisms of stress tolerance promoted by bio-stimulants. It has been shown that bio-stimulants interfere with the expression of CS responsive transcription factors, photosynthesis-related genes, antioxidant enzymes, and heat shock proteins, which are involved in different functional areas of cold response in a plant. Transcriptomic research offers informative indications that bio-stimulants have extreme impacts on gene expression webs controlling the response to cold stress, mainly by alteration of cold-responsive transcription factors (TFs) and signaling elements (Ali et al., 2024).

The CBF/DREB regulon is a bean mediator that regulates the cold stress response in tobacco (Caccialupi et al., 2023). These transcription factors were found to induce expression of several cold-induced genes, like enzyme coding genes that scavenge ROS, dehydrins, protein-coding genes that neutralize ROS, and the late embryogenesis abundant proteins (LEA) that play a role in protection from freezing stress (Wu et al., 2024). In keeping with this, a transcriptome profiling identified highly increased expression of known master genes of cold acclimation when using the microbial products, seaweed extracts, and humic acids, which are typical of the CBF gene family (CBF1, CBF2, and CBF3 as well as the DREB gene family, DREB1A and DREB2A; Ucar et al., 2026). For example, when ANE was applied to *A. thaliana*, CBF transcripts were significantly increased after 12 h at 4 °C, and transcripts of cold-regulated genes (e.g., COR15A, COR47, and RD29A), which are involved in membrane stabilization and osmotic adjustment, significantly increased (Gusain et al., 2023; Qian et al., 2024). Similarly, showed that ANE-treated tomato plants subjected to CS displayed transcriptional reprogramming of CBF pathway genes (Fang et al., 2021; Yun et al., 2022). Moreover, the activation of heat shock transcription factors (HSFs), emphasizing the interaction between cold and heat stress signaling pathways, is facilitated by bio-stimulants (Varun and Lakshmi, 2025).

Bio-stimulants also affect transcription factors regulated by ABA, such as the AREB/ABF family involved in stomatal closure, osmotic balance, and antioxidant production (Wei et al., 2021). Application of humic acid-based bio-stimulant led to a significant rise in the expression of AREB1 and ABF3 and activation of genes coding for late embryogenesis abundant proteins and osmoprotective sugar in rich seedlings under chilling stress (Ramakrishnan et al., 2022). From this, it can be concluded that the phytohormones are acting in concert with the cold signaling pathway, all while maintaining low levels of data transfer (Canellas et al., 2022). This means bio-stimulants effectively use both hormonal and cold signaling routes and strengthen their adaptive signals in an efficient manner, while keeping data transfer low (Khatab et al., 2022; Kaleh et al., 2025). Furthermore, it can be stated that bio-stimulants activate heat shock transcription factors (HSFs), thereby revealing an interesting interplay between cold stress signaling and heat stress signaling (Zahid et al., 2026). Microbial origin bio-stimulants, such as plant growth-promoting rhizobacteria (PGPR), have been shown to affect transcription factors of ethylene and jasmonic acid gene regulation systems as a way of enhancing cold tolerance by providing antioxidant cell protection and cell wall reinforcement (Anli et al., 2020).

One of the most consistent and obvious transcriptional responses to bio-stimulation stress under cold conditions is activation of antioxidant gene groups like APX, SOD, CAT, and GST, showing a decrease in ROS accumulation and other oxidative stress effects (Varadharajan et al., 2025). Bio-stimulants have been shown to induce higher expression of the proteins of stress, including HSP70 and HSP90, in tobacco plants and thus stabilize the proteins in the cell during dehydration stress (Hu et al., 2022). Additionally, RNA-Seq data indicate that the genes involved in sugar metabolism and energy signaling, such as those involved in the control of osmotic responses to cold stress, are affected by bio-stimulant treatments (Chen et al., 2024b). Interestingly, bio-stimulants also act on a post-transcriptional regulatory level involving microRNAs (miRNAs) as regulators of transcription factors (Junaid et al., 2024). The whole set of these genomic findings collectively indicates that bio-stimulants are effective as modulators for gene expression to initiate a multi-faceted transcriptional defense network, such as TFs, hormonal regulators, antioxidant enzymes, and biosynthesis of osmoprotectant, with the view of enhancing cold tolerance (Wang et al., 2025).

Proteomics is now a viable technique to reveal the complex molecular responses underlying bio-stimulant-mediated cold stress tolerance in plants (Varadharajan et al., 2025). In contrast, proteomic profiling does not predict potential changes, as it is also a transcriptomic technique, but provides direct evidence of operational proteins, which are involved in the process of stress adaptation (Li et al., 2024b). Proteomic analyses have identified specific proteins modulated by bio-stimulants under cold stress (summarized in Supplementary Table S1). Cold stress disrupts protein stabilization, folding, and cellular homeostasis and leads to metabolic defects and oxidative damage. Bio-stimulants control these effects by modifying the amounts, stability, and activity of specific groups of proteins associated with stress resistance (Agho et al., 2025). Its analysis indicated that HSPs and molecular chaperones can dramatically increase with bio-stimulant treatment, and play a fundamental role in preserving protein conformation at CS (Raza et al., 2023). HSP70 helps to refold proteins that are denatured, and HSP90 stabilizes signaling proteins. This rise of chaperones is linked to improved photochemical potential and stability of chloroplasts in plants being exposed to seaweed extracts or humic compounds during stress. Bio-stimulants have a positive effect on photosynthetic proteins such as photosystem II and the Calvin cycle. Cold stress decreases electron transportation, whereas in plants that are treated, the quantities of Rubisco are activated and other vital proteins are maintained that enabling the carriage out of carbon (Bhattacharya, 2022). In wheat seedlings, humic acid improves HSP levels and stabilizes PSII proteins, which help trap Energy and protect against photohyperoxides (Thirumal, 2024).

The activation of antioxidant defense proteins has become another critically important aspect in protein omics, as it witnesses the response to oxidative stress generated by the excess amount of reactive oxygen species (ROS) in cold conditions (Ali et al., 2022). The reaction of proteins like superoxide dismutase (SOD), catalase (CAT), ascorbate peroxidase (APX), and glutathione S-transferase (GST) drastically increases after the bio-stimulant is used (Sheri et al., 2023). They inhibit lipid peroxidation and preserve a membrane, thereby neutralizing ROS like hydrogen peroxide and superoxide radicals. Higher concentrations of these enzymes are associated with reduced amounts of malondialdehyde (MDA), a marker of oxidative damage in treated plants (Ding et al., 2023). Sensitivity proteomic analyses identified bio-stimulants to play roles in signal transduction proteins and stress-relevant kinases, including MAPKs and CDPKs. To demonstrate, *Ascophyllum nodosum* extract also triggered MAPK cascades, leading to the activation of ABA-sensitive proteins that enhance water use efficiency in cold-induced dehydration stress (Ali et al., 2021). Proteomic data indicate bio-stimulants use in the response to chilling stress results in the activation of previously dormant enzymes that catalyze flavonoid and lignin synthesis, cell wall strengthening, and antioxidant response enhancements downloads through significant changes in levels of such well-known enzymes as phenylalanine ammonia-lyase (Koźmińska et al., 2026). This evidence explains that bio-stimulants improve cold tolerance by stabilizing protein, promoting antioxidant defense, promoting signaling pathway activation, and promoting metabolic plasticity. These results and other transcriptomic and metabolomics data reveal the value of sophisticated quantitative proteomics in the study of bio-stimulant impacts across crops as part of precision agriculture in the face of climate variations.

Metabolomics studies have revealed marked changes in the trend of osmolytes and secondary metabolites after bio-stimulant interventions (Ben Mrid et al., 2021). The presence of osmolytes that increase cold stress, like proline and trehalose, enhances cell stability. An almost 60% increment in proline was observed in maize under chilling stress; fellow researchers (Singh et al., 2024) found increased maize trehalose under seaweed extracts. Secondary metabolites increase with improved ROS-elimination and cell defense mechanisms. As shown by Amin et al. (2024), the levels of flavonoids in cucumber leaves treated with protein hydrolysates during cold stress led to a subsequent improvement of antioxidant capacity and photosynthetic efficiency, but in (Meggio et al., 2020), the levels of phenolics in grapevines treated with protein hydrolysates after freezing were significantly increased. More recent studies have added to these results some quantitative support: humic acid application in melon seedlings facing cold stress raised chlorophyll levels by 33.17%, enhanced Fv/Fm ratios, and increased the activities of nitrogen metabolism enzymes including nitrate reductase (NR), glutamine synthetase (GS), glutamate synthase (GOGAT), and glutamate dehydrogenase (GDH) by as much as 181.83%, in addition to an increase in proline biosynthesis enzymes P5CS and OAT by up to 81.97%, collectively improving nitrogen uptake and osmotic regulation (Zhu et al., 2024). Similarly, tomatoes treated with seaweed extracts possessed milder cold stress conditions due to the processing of photosynthetic pigments, the reduction of H₂O_2_ and MDA, and the strong rise in proline contents, accompanied by an impressive boost in antioxidant enzymes, including glutathione S-transferase (GST), peroxidase (POX), and SOD (Chanthini et al., 2023). Besides these physiological and biochemical responses, emerging evidence suggests that bio-stimulants could also modulate interactions of root-microbiomes under stressful conditions, which has an indirect benefit of cold tolerance (Liu et al., 2022). Activities of microbes within the rhizosphere improve nutrient acquisition and phytohormonal signaling, thereby supporting plant fitness during harsh environments (Chang et al., 2020). Integrating multi-omics datasets reveals that bio-stimulant-mediated tolerance involves a complex signaling network with increased ABA biosynthetic genes (12-fold), antioxidant gene induction (40-70-fold), improved enzymatic activities (30–180%), and enhanced chlorophyll and proline content (30–60%) (Kong and Liu, 2022). These molecular changes correlate with improved biomass, photosystem stability, and reproductive success in various crops under cold stress, confirming bio-stimulants’ systems-level impact. Research indicates the significance of protein hydrolysates, humic acids, and microbial products when used alongside seaweed-derived products in alleviating abiotic stress. Recent methodological progress (primarily multi-omics techniques and machine learning algorithms) has accelerated the creation of bio-stimulants, an essential component of precision agriculture and crop resilience against climate change.

## 9 Future prospects

Future research should focus on hypothesis-driven research that clarifies how improvements in cold stress resilience in tobacco occur at the bio-stimulant’s molecular and biochemical level. Comparative field trials are necessary to evaluate the effectiveness of microbial and non-microbial bio-stimulants, including arbuscular mycorrhizal fungi (AMF) and plant growth-promoting rhizobacteria (PGPR) as compared with the non-microbial bio-stimulants such as seaweed extracts and humic substances. A field trial could be conducted on contrasting effects of AMF and seaweed extract after 7 days of 5 °C cold exposure in terms of photosynthetic efficiency, antioxidant enzyme activity, biomass production, leaf quality etc. Further, optimization of dosage and application technique of bio-stimulants is another important research area. Research should be focused on examining the benefits of seed priming, foliar spraying, and multi-stage applications of both methods at different growth stages for improving cold tolerance. Ideally, a testable hypothesis should be that this dual application (seeding and foliar spraying) provides superior protection because the physiological and molecular responses are increased as compared to treatments made just to the seed and just to the foliar surface. In further research, the incorporation of transcriptomics and proteomics along with metabolomics, can be used to find the key regulatory networks that are involved in stress adjustment induced by bio-stimulants. Treated plants may be proposed to have a higher expression of the CBF/COR genes, a higher concentration of antioxidant proteins and higher concentration of osmoprotectant metabolites than untreated plants in longer, extended chilling situations.

## 10 Concluding remarks

Bio-stimulants are now proven tools to help alleviate the effects of cold stress on tobacco and enhance various processes, including physiological function, antioxidant defense, photosynthetic efficiency, nutrient uptake, and stress response molecular pathways. Microbial and non-microbial bio-stimulants boost the resilience of plants, orchestrate hormonal signaling, osmotic adjustment, and metabolic reprogramming, which help plants cope with low-temperature environments. Recent developments in transcriptomics, proteomics and metabolomics have shed light on the molecular mechanisms of these responses and identified potential molecules as markers for developing strategies that make cultivation of tobacco more resilient to climate change. Although these are promising results, bio-stimulants are not necessarily effective. These have a multiple effect depending on the interaction between various factors including tobacco genotype, environmental factors, stress level, bio-stimulant type, timing, and dosage. The effects of a bio-stimulant may also be different in various cultivars and environments, and hence, this needs to be adjusted on a case-by-case basis and not assumed to work in general. The environmental impacts of bio-stimulants are typically less than those of synthetic agrochemicals, and there is no life cycle assessment (LCA) data that is specific to tobacco production under cold stress. To conclude, the environmental safety of bio-stimulant formulations, energy inputs, production techniques, soil persistence, and environmental impacts should be assessed in the future. Future studies should focus on field trials to be repeated, ideally employing a diversity of classes of bio-stimulants, and on studies to characterize and break down the central regulatory network and stable markers of cold tolerance. Improved prediction and optimization of bio-stimulant use in various scenarios will be possible thanks to precision agriculture technologies and high throughput phenotyping, and AI data analysis. It can be concluded, therefore, that bio-stimulants might be another solution to developing more cold tolerant tobacco plants; however, the product needs to be optimized for the specific varieties and conditions. Further research should be directed towards genotype-specific evaluations, field evaluation of the outcomes, and towards strategies for combining applications to enhance the benefits of the crops under the changing climate.

## 11 Testable hypotheses for future research

1. Foliar application of *Ascophyllum nodosum* extract upregulates NtCBF1 and NtICE1 transcript levels within 6 h of cold exposure (4 °C) in tobacco leaves
2. Combined application of PGPR (*Bacillus subtilis* GB03) and protein hydrolysate produces synergistic effects on SOD and APX activities, resulting in >50% reduction in malondialdehyde (MDA) compared to single treatments
3. Cold-tolerant and cold-sensitive tobacco genotypes exhibit differential antioxidant gene expression (NtSOD, NtAPX1, and NtCAT1) responses to identical bio-stimulant regimens under chilling stress.
4. Combined bio-stimulant application (seaweed extract + humic acid + PGPR) produces greater cold tolerance than any single bio-stimulant through activation of multiple complementary pathways.
5. Bio-stimulant-mediated cold tolerance is associated with specific shifts in rhizosphere bacterial communities, including increased relative abundance of Flavobacterium and Arthrobacter
6. Precision application of bio-stimulants guided by real-time chlorophyll fluorescence (Fv/Fm) monitoring reduces required dosage by ≥30% while maintaining cold protection efficacy.
