# Supplementary material for: Bio-stimulants enhance cold tolerance in tobacco through antioxidant defense and photosynthetic regulation
Source: Front Microbiol. 2026 Jul 20;17:1884028. doi: 10.3389/fmicb.2026.1884028 (PMC13448450; doi:10.3389/fmicb.2026.1884028)
Supplement: Supplementary file 1 [file Table_1.DOCX]

| **Protein name** | **Plant species** | **Bio-stimulant treatment** | **Fold-change** | **Reported effect under cold stress** | **Reference** |
| --- | --- | --- | --- | --- | --- |
| HSP70 | *Arabidopsis thaliana* | Humic acid / PGPR | +2.3 | Enhanced protein folding & stress protection | (Ahmad et al., 2022) |
| Rubisco activase | *Nicotiana tabacum* | Seaweed extract | +1.8 | Improved photosynthetic efficiency | (Tian et al., 2025) |
| APX1 (Ascorbate peroxidase 1) | *Solanum lycopersicum* | Chitosan treatment | +3.1 | Reduced H₂O₂ accumulation, improved ROS scavenging | (El Amerany et al., 2022) |
| Fe-SOD (Iron superoxide dismutase) | *Zea mays* | PGPR inoculation | +2.5 | Enhanced antioxidant defense under chilling stress | (Borella et al., 2023) |
| CAT (Catalase) | *Oryza sativa* | Seaweed extract | +2.0 | Improved hydrogen peroxide detoxification | (Wu et al., 2022) |
| LEA proteins | *Triticum aestivum* | Humic substances | +2.7 | Stabilization of cellular structures under cold stress | (Rawal et al., 2025) |
| PsbA (D1 protein of PSII) | *Arabidopsis thaliana* | Biochar + PGPR | +1.6 | Protection of photosystem II under low temperature | (Parastesh et al., 2025) |

Table S1: Key proteins modulated by bio-stimulants in plants under cold stress: proteomic evidence
